# Proton Therapy for Major Salivary Gland Cancer: Clinical Outcomes

**DOI:** 10.14338/IJPT-20-00044.1

**Published:** 2021-06-25

**Authors:** Alexander N. Hanania, Xiaodong Zhang, G. Brandon Gunn, David I. Rosenthal, Adam S. Garden, C. David Fuller, Jack Phan, Jay P. Reddy, Amy Moreno, Gregory Chronowski, Shalin Shah, Noveen Ausat, Ehab Hanna, Renata Ferrarotto, Steven J. Frank

**Affiliations:** 1 Department of Radiation Oncology, Baylor College of Medicine, Houston, TX, USA; 2 Department of Radiation Oncology, The University of Texas MD Anderson Cancer Center, Houston, TX, USA; 3 Department of Radiation Physics, The University of Texas MD Anderson Cancer Center, Houston, TX, USA; 4 Department of Head and Neck Surgical Oncology, The University of Texas MD Anderson Cancer Center, Houston, TX, USA; 5 Department of Head and Neck Medical Oncology, The University of Texas MD Anderson Cancer Center, Houston, TX, USA

**Keywords:** proton therapy, major salivary gland cancer, unilateral, toxicity, dermatitis

## Abstract

**Purpose:**

To report clinical outcomes in terms of disease control and toxicity in patients with major salivary gland cancers (SGCs) treated with proton beam therapy.

**Materials and Methods:**

Clinical and dosimetric characteristics of patients with SGCs treated from August 2011 to February 2020 on an observational, prospective, single-institution protocol were abstracted. Local control and overall survival were calculated by the Kaplan-Meier method. During radiation, weekly assessments of toxicity were obtained, and for patients with ≥ 90 days of follow-up, late toxicity was assessed.

**Results:**

Seventy-two patients were identified. Median age was 54 years (range, 23-87 years). Sixty-three patients (88%) received postoperative therapy, and nine patients (12%) were treated definitively. Twenty-six patients (36%) received concurrent chemotherapy. Nine patients (12%) had received prior radiation. All (99%) but one patient received unilateral treatment with a median dose of 64 GyRBE (relative biological effectiveness) (interquartile range [IQR], 60-66), and 53 patients (74%) received intensity-modulated proton therapy with either single-field or multifield optimization. The median follow-up time was 30 months. Two-year local control and overall survival rates were 96% (95% confidence interval [CI] 85%-99%) and 89% (95% CI 76%-95%], respectively. Radiation dermatitis was the predominant grade-3 toxicity (seen in 21% [n = 15] of the patients), and grade ≥ 2 mucositis was rare (14%; n = 10 patients). No late-grade ≥ 3 toxicities were reported.

**Conclusion:**

Proton beam therapy for treatment of major SGCs manifests in low rates of acute mucosal toxicity. In addition, the current data suggest a high rate of local control and minimal late toxicity.

## Introduction

Radiation therapy (RT), usually delivered postoperatively, remains a cornerstone in the treatment of major salivary gland cancers (SGCs). These tumors are unique, relatively rare, and heterogenous in histology and behavior [1]. Despite the lack of randomized control trials, it is widely accepted that, in the setting of high-risk disease features, RT improves local and regional control for these cancers, and this has been demonstrated in retrospective series over decades with both conventional RT and intensity-modulated radiation therapy (IMRT) [2–5]. The benefits of RT, however, must be balanced with its associated toxicities. Fortunately, and secondary to anatomic location (parotid and submandibular) and nodal drainage, most major SGCs can be treated with unilateral radiation. Nevertheless, the oral cavity and contralateral salivary glands remain at risk, with rates of grade 2 or higher mucositis—a surrogate for minor salivary gland dose—as high as 77% and acute xerostomia as high as 62%, despite the use of IMRT [6]. Alternatively, proton beam therapy (PBT) may provide an attractive solution for mucosal toxicity.

Data continue to emerge defining the dose-sparing potential of PBT for head and neck cancers. For major SGCs and other unilateral head and neck treatments, the immediate falloff of dose theoretically preserves the oral cavity and sublingual and contralateral salivary glands. Until recently, passively scattered proton therapy (PSPT) had been the most widely adopted form of PBT. Although PSPT allows sparing of structures distally, the inability to appropriately sculpt the beam and conform it around both proximal and distal structures remains. Intensity-modulated proton therapy (IMPT) has led to improvements in planning. Robust single-field and multifield optimization (SFO/MFO) allow treatment of complex volumes and maintenance of dosimetric equipoise in surrounding structures at both the proximal and distal extent [7, 8]. From a biologic perspective, patients with radioresistant tumors that are prone to recurrence may also stand to benefit from future advances in IMPT that allow linear energy transfer and relative biological effectiveness (RBE)–optimized radiation planning [9]. Further, the ability to safely reirradiate (with cumulative doses) is made possible with advancements in particle therapy [2, 10–12].

Although the theoretical benefits are clear, clinical outcomes data for head and neck subsites are needed to establish the safety and clinical efficacy of PBT. Therefore, in the current study, we report our clinical outcomes in terms of disease control and prospectively assessed toxicities for patients with SGCs treated with PBT.

## Materials and Methods

### Patient Selection and Enrollment

All patients with head and neck cancer treated with PBT at our institution are enrolled in an institutional review board–approved, prospective, observational protocol (PA11-0803, PCR05-0207). Data are collected during and after the PBT and include weekly assessments of acute treatment toxicity. The protocol permits analysis of pretreatment patient and disease characteristics, time to local and distant progression, and survival. From this large cohort of patients, we identified those who had definitive or postoperative PBT for SGC from August 2011 to February 2020 for data extraction. Because one of the principal endpoints of the study was to assess toxicity to healthy tissues, patients with squamous cell carcinoma involving the parotid gland and patients with confirmed or suspected oligometastatic disease were included. Clinical and dosimetric characteristics, including: age, sex, smoking history, salivary gland subsite, stage per *American Joint Committee on Cancer Staging Manual*, 8th edition [13], histology, margin status, presence of perineural involvement and/or extranodal extension, treatment sequence, initial or recurrent disease status, delivered dose, number of fractions, inclusion of ipsilateral neck (levels IA-V) in RT fields, and use of concurrent chemotherapy were abstracted. Dosimetric details were obtained from the treatment planning system for the approved and delivered plan, and the number of plan adaptations required for each patient was assessed.

### Treatment Planning and Delivery

All patients met treatment indications for adjuvant or definitive radiation per the National Comprehensive Cancer Network (NCCN, Fort Washington, Pennsylvania) and institutional treatment guidelines and multidisciplinary tumor-board discussion. In general, patients underwent computed tomography simulation with a 5-point thermoplastic mask as an immobilization aid with target delineation. A tissue-equivalent bolus was not used; however, for postoperative patients, the entirety of the surgical scar was included in the clinical treatment volume. Treatment of the ipsilateral neck was per discretion of the treating radiation oncologist based on risk (eg, high-grade histology, cervical nodal metastasis). In cases of facial nerve involvement, coverage of the facial canal is institutional practice. Other named nerve involvements are followed to the base of skull (eg, a branch of the trigeminal nerve would be followed to the foramen). Standard postoperative dose was 60 Gy. Consideration of a 4- to 6-Gy boost was given for patients with close or positive margins and/or extraglandular extension and in cases of resected nodal metastases with extracapsular extension. For patients with unresectable or gross disease, standard dose was 70 Gy. Elective nodal irradiation, when indicated, was covered to 50 to 57 Gy, depending on fractionation and risk. Image guidance included daily kilovoltage imaging, and verification simulations were performed during weeks 1 and 3 per practice. Adaptive plans were used when clinically observed deviations from plan intent were noted.

**Figure 1.**
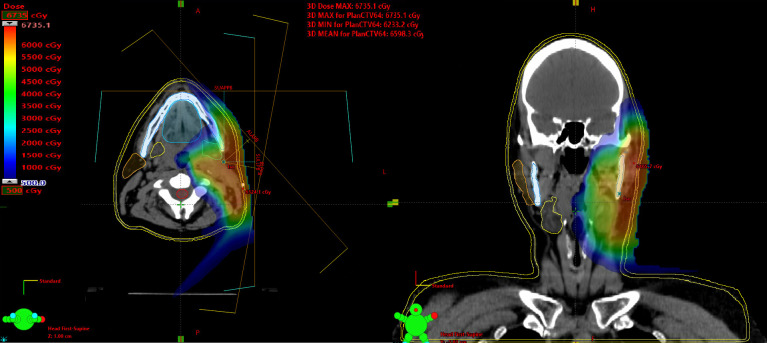
Proton therapy plan for unilateral treatment of salivary gland cancer. Presented is an example of an intensity-modulated proton therapy plan with multifield optimization, and the dose is displayed in color wash on the axial and coronal planes. Blue represents the minimum dose displayed at 500 (cGyRBE), with red at or above the prescription dose. In terms of organs at risk, at midline, the oral cavity is contoured (teal) as well as the spinal cord (red); on the contralateral side, the parotid (orange) and submandibular gland (yellow) are contoured. Proton therapy allows for marked sparing of those structures from clinically significant doses of radiation.

Per institutional standards, scanning beam delivery was preferred for complex scenarios and for cases in which multiple dose tiers were required. In addition, PSPT was used for some patients with head and neck (HN) disease; however, PSPT was generally restricted to scenarios with uniform, nonconcave clinical-target volumes. Initially, we used SFO for unilateral treatment, but more recently, given the development of robust optimization, we have further adopted MFO for unilateral treatment. The Eclipse treatment planning system (Varian Medical Systems, Palo Alto, California) was used to calculate all plans for delivering proton therapy with discrete spot-beam scanning. The finalized IMPT plan was then delivered by a synchrotron and a proBEAT PBT system (Hitachi, Ltd, Tokyo, Japan). In general, 2- or 3-beam arrangements were used for unilateral HN treatment. Currently, our class solution for unilateral HN plans includes a standard 3-field beam arrangement involving 2 right or left anterior oblique beams and a posterior-anterior beam. The beams are noncoplanar, and robustness is considered (Figure 1).

### Clinical Assessment and Follow-Up

The Common Terminology Criteria for Adverse Events, version 4 (CTCAEv4), was used for acute and late toxicity assessments. After treatment, patients were followed by policy at 3- to 6-month intervals, per NCCN recommendations, with surveillance imaging per an algorithmic follow-up policy. *Local failure* was defined as clinical or radiographic evidence of progression or recurrence at the primary tumor site (in the parotid or submandibular gland or in the postoperative bed) or within a treated neck volume and was further classified per institutional standards [14]. *Progression* was defined to include local failure, regional failure (defined as outside the treated volume), development of distant metastatic disease, and death. Further, patients were referred for baseline and continued audiometry monitoring studies, which were available for analysis in 41 of the 72 patients (57%). Patients with < 90 days of follow-up were excluded from analysis of late toxicities. Patients traveling to our clinic for PBT but returning to their home institution for continued care were sent questionnaires mailed at 6-month intervals to follow up on the patients' oncologic outcome, and outside records were requested to retrospectively assess any late toxicities.

### Statistical Analysis and Dosimetric Review

Local control and overall survival analyses were performed by the Kaplan-Meier method with Prism software (version 8.0.0 for Windows, GraphPad Software, San Diego, California). Confidence intervals are reported at the 95% level. For local control analyses, patients were censored at time of death. Progression-free survival was calculated for a subset of patients, excluding patients with regionally metastatic squamous cell carcinoma (ie, from a cutaneous primary) or oligometastatic disease and included freedom from death. Summary statistics of categorical and continuous variables were computed with JMP software (version 14, SAS Institute Inc, Cary, North Carolina). Organs-at-risk were contoured per accepted consensus-delineation guidelines [15]; however, oral cavity contours did not purposefully include the anterior oropharynx, as is sometimes done with an “extended” oral-cavity contour [15]. Standard dosimetric variables abstracted for analysis included the highest prescribed target-volume dose, the number of fractions, the maximum point dose (percentage), and the proton delivery technique (SFO, MFO, or PSPT). For organs at risk, mean dose to the oral cavity and contralateral submandibular and parotid glands as well as the maximum dose to the brain stem, spinal cord, and skin were recorded in GyRBE. Of note, dose to the ipsilateral submandibular gland was abstracted through post hoc contouring for patients in which the gland was intact and not part of the clinical target volume (CTV; parotid cases not requiring treatment of level IB) but was not used as an avoidance structure during treatment planning. Finally, to assess coverage, the minimum dose to 95% of the CTV (CTV 95) was also examined.

## Results

### Patient, Disease, and Treatment Characteristics

Seventy-two patients were included. The median age was 54 years (range, 23-87 years), and the most common histologies included mucoepidermoid (21%; n = 15) and adenoid cystic carcinoma (18%; n = 13). Most patients (88%; n = 63) were treated with adjuvant therapy, and more than one third of patients (36%; n = 26) received concurrent chemotherapy. Nine patients (12%) were treated in the reirradiation setting. Four patients (6%) had oligometastatic disease (a single lesion in each case). All patients except one (99%; n = 71) who presented with bilateral adenopathy at diagnosis, received unilateral treatment.

The median dose was 64 GyRBE (range, 56-70 GyRBE) overall, 70 GyRBE (range, 64-70 GyRBE) for definitive treatment, and 60 GyRBE (range, 56-70 GyRBE) for postoperative adjuvant treatment. In terms of the ipsilateral neck, level II was treated in 78% (n = 56 patients) of the cases; level III, in 62% (n = 45); level IV, in 36% (n = 26); level IB, in 42% (n = 30); level V, in 4% (n = 3); and level IA, in 3% (n = 2). The median elective neck dose was 54 GyRBE (range, 46-57 GyRBE). In addition, IMPT was used in 53 cases, of which, 60% (n = 32) were planned with SFO and 40% (n = 21) were planned with MFO. Of the nine patients (12%) who underwent reirradiation of the HN, prior RT had been given 1 to 20 years earlier, with all cases having received prior doses of ≥ 50 Gy, except in 1 case of mantle RT for lymphoma with a submandibular boost (approximately 40 Gy). Dose was not intentionally reduced in any of those cases; however, cumulative dose was taken into consideration, as well as the volumes treated, and thus only 2 (22%) of these 9 cases included elective coverage of ipsilateral neck levels. Only 1 patient (1%) terminated treatment early (at 27 of 30 fractions), secondary to fatigue. Patient, disease, and treatment characteristics are presented in Table 1.

**Table 1.** Clinical and disease characteristics; N = 72 patients.

**Factor**	**No. (%)**
Age, y	
20-39	16 (22)
40-59	26 (36)
≥ 60	30 (42)
Sex	
Male	41 (57)
Female	31 (43)
Smoking history	
Yes	9 (13)
No	63 (87)
Subsite	
Parotid	66 (92)
Submandibular	6 (8)
Histology	
Mucoepidermoid	15 (21)
Adenoid cystic	13 (18)
Acinic cell	9 (12)
Squamous cell carcinoma	9 (12)
Carcinoma ex pleomorphic adenoma	8 (11)
Adenocarcinoma (not otherwise specified)	5 (7)
Salivary duct carcinoma	5 (7)
Other	8 (11)
AJCC 8th Edition [13] stage	
Primary cases (n = 53)*	
I	18 (34)
II	7 (13)
III	13 (25)
IV (A/B/C)	15 (28)
Recurrent cases (n = 19)	
Treatment details (n = 72)	
Definitive	9 (12)
Adjuvant	63 (88)
Positive margin	25 (40)
Concurrent chemotherapy	26 (36)
Node positive	18 (25)
Extranodal extension	8 (11)
Perineural involvement	25 (35)
Named nerve	3 (4)
Prior HN radiation	9 (12)

**Abbreviations:** AJCC, American Joint Committee on Cancer*;* HN, head and neck.

* Initial treatment course (ie, nonrecurrent disease).

Overall, the median oral cavity mean dose was 2.4 GyRBE, and the median brainstem mean dose was 4.2 GyRBE. Six patients (8%) with submandibular gland primaries received a median oral cavity mean dose of 8.3 GyRBE (interquartile range [IQR], 2.6-10.8 GyRBE). Dose to the contralateral parotid and submandibular glands approached zero. For cases not requiring elective RT of level IB and with an intact ipsilateral submandibular gland, the median mean dose to that structure was 47.6 GyRBE (IQR, 38.0-54.8 GyRBE). For all cases, CTV D95 exceeded the prescription dose. Further dosimetric treatment characteristics are presented in Table 2**.**

**Table 2.** Dosimetric details.

**Parameter**	**Value**
Treatment variable	
Rx dose, median (IQR), GyRBE	64 (60-66)
Maximum point dose, median (IQR)	111 (109-113)
Coverage (CTV D95%/Rx dose), median (IQR), %	101% (101%-102%)
Technique, n (%)	
Passively scattered proton therapy (PSPT)	19 (26)
Intensity modulated proton therapy (IMPT)	53 (74)
Single-field optimization (SFO)	32 (60)
Multifield optimization (MFO)	21 (40)
Organs at risk, median (IQR), GyRBE	
Mean contralateral parotid gland	0 (0-0)
Mean contralateral submandibular gland	0.03 (0-0.1)
Mean oral cavity	2.4 (1.0-5.8)
Brainstem maximum dose	4.2 (3.9-14.6)
Spinal cord maximum dose	5.5 (0.6-12.4)

**Abbreviations:** Rx, prescription; IQR, interquartile range; GyRBE, Gy radiobiological equivalent; CTV, clinical target volume.

### Local Control and Progression-Free and Overall Survival

**Figure 2.**
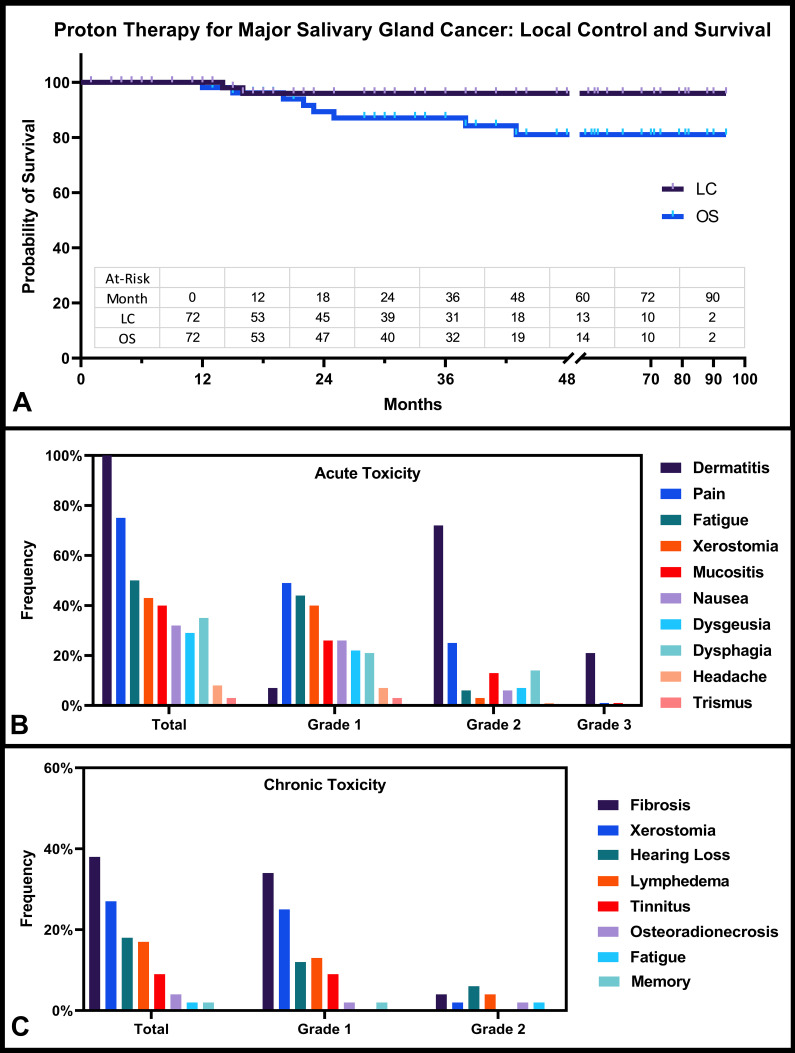
Clinical outcomes of proton therapy for salivary gland cancer. Presented are the (A) Kaplan-Meier curves for local control (LC) and overall survival (OS) for the entire cohort. (B) Prospectively recorded acute toxicity rates per Common Terminology Criteria for Adverse Events, version 4 (CTCAEv4) are reported for all 72 patients. (C) Retrospectively assessed chronic toxicities for the 53 patients with > 90 days of follow-up are shown.

The median follow-up time was 30 months (range, 3-94 months) with 7 patients (10%) lost to follow-up. Survival curves are presented in **Figure 2A**. For the entire cohort, 2-year local control and overall survival rates were 96% (95% confidence interval [CI] 85%-99%) and 89% (95% CI 76%-95%), respectively. At 5 years, the actuarial local control and overall survival rates were estimated at 96% (95% CI 85%-99%) and 81% (95% CI 66%-90%), respectively. At the time of last follow-up, 2 patients (3%) had local failure, as previously defined, one of which was a central high-dose failure (type A), and the other was a central elective-dose failure (type C-low). In a subset analysis (n = 60; 83%) that excluded patients with metastatic squamous cell carcinoma and oligometastatic disease, the 2-year progression-free survival rate was 77% (95% CI 62%-87%). Within that subset, 1 patient (2%) developed regional metastatic disease outside the treated field (hard palate and clivus), and 8 patients (13%) developed distant metastatic disease (lungs, 56% [n = 5]; bone, 33% [n = 3]; liver, 22% [n = 2]; and brain, 22% [n = 2]).

### Acute and Late Toxicity

**Figure 3.**
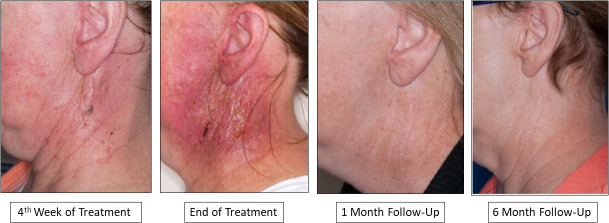
Clinically significant dermatitis and medical photography evaluation. An example is presented of a patient in the study who developed clinically significant dermatitis, which was documented with medical photography during and after treatment. Grade 3 dermatitis, defined per the Common Terminology Criteria for Adverse Events (CTCAEv4), included moist desquamation in areas outside of the skin folds as well as the possibility of bleeding secondary to minor abrasions or trauma. In our study, 93% of patients (n=67) developed grade ≥ 2 dermatitis, whereas 21% (n = 15) developed grade 3 dermatitis.

Acute and late toxicities are reported in **Figure 2B** and **2C** and in Table 3. Grade 3 radiation dermatitis was observed in 21% (n = 15) of the patients (**Figure 3)**. There was only 1 case (1%) of grade 3 mucositis. Grade 2 or higher recorded acute toxicity per the CTCAEv4 was seen for dermatitis (93%; n = 67), followed by pain (26%; n = 19), mucositis (14%; n = 10), dysphagia (14%; n = 10), dysgeusia (7%; n = 5), fatigue (6%; n = 4), nausea and/or vomiting (6%; n = 4), and xerostomia (3%; n = 2). No patient required a feeding tube because of PBT (1 patient presented to PBT with a tube required because of the extent of surgical resection). The 6 patients (8%) with submandibular gland tumors did not appear to exhibit significantly different acute toxicities; dermatitis was the sole grade 2 or higher toxicity among this subset.

**Table 3.** Acute and chronic toxicities.

**Toxicities**	**Grade 1, No. %**	**Grade 2, No. %**	**Grade 3, No. %**	**Total, No. %**
Acute (n = 72)				
Dermatitis	5 (7)	52 (72)	15 (21)	72 (100)
Pain	35 (49)	18 (25)	1 (1)	54 (75)
Fatigue	32 (44)	4 (6)	0	36 (50)
Xerostomia	29 (40)	2 (3)	0	31 (43)
Mucositis	19 (26)	9 (12)	1 (1)	29 (40)
Nausea/vomiting	19 (26)	4 (6)	0	23 (32)
Dysgeusia	16 (22)	5 (7)	0	21 (29)
Dysphagia	15 (21)	10 (14)	0	25 (35)
Headache	5 (7)	1 (1)	0	6 (8)
Trismus	2 (3)	0	0	2 (3)
Chronic (n = 53)				
Fibrosis*	18 (34)	2 (4)	0	20 (38)
Xerostomia	13 (25)	1 (2)	0	14 (26)
Lymphedema	7 (13)	2 (4)	0	9 (17)
Hearing loss	6 (11)	3 (6)	0	9 (17)
Tinnitus	5 (9)	0	0	5 (9)
Osteoradionecrosis	1 (2)	1 (2)	0	2 (4)
Fatigue	0 (0)	1 (2)	0	1 (2)
Memory	1 (2)	0	0	1 (2)

* Includes skin and/or soft tissue of the neck.

Late toxicity was assessed collectively for 53 patients (74%). At a median follow-up time of 41 months, no grade ≥ 3 late toxicities were reported. The most common grade 2 late toxicities included hearing loss (6%; n = 3), fibrosis (4%; n = 2), and lymphedema (4%; n = 2). Significant late xerostomia (grade ≥ 2) was seen in a single patient (2%), and there were no reports of late dysgeusia. Of the 9 patients (17%) developing grade ≥ 1 hearing loss, 44% (n = 4) had received concurrent chemotherapy; all 9 patients had ipsilateral hearing loss, and 33% (n = 3) had bilateral hearing loss. Two cases (4%) of osteoradionecrosis were reported, one (asymptomatic) found incidentally on follow-up magnetic resonance imaging and the other (grade 2) managed with close dental evaluations and debridement.

## Discussion

In the current study, and after nearly a decade of prospective data collection, our results demonstrate that PBT for major SGCs is associated with low rates of acute mucosal toxicity. Clinically significant dermatitis was seen in nearly all enrolled patients, which is to be expected given that most patients are treated postoperatively and that the surgical scar was included in the prescription dose. Further, 2-year control and survival rates were high, and no grade 3 or higher late toxicities have been reported. Our study expands on the current literature because we report not only acute toxicity but also disease control, late toxicity, and comprehensive dosimetry details. Further, because much of the previous data have been on PSPT, our cohort is unique in that most patients (53, 74%) were treated with IMPT.

In terms of PBT versus IMRT, a comparative study for unilateral HN treatments, found that PBT significantly reduced the maximum brainstem dose (from about 30 Gy to < 1 Gy), mean oral cavity dose (from about 21 Gy to < 1 Gy), mean contralateral parotid dose (1.4 Gy to 0 Gy), and mean contralateral submandibular gland dose (4.1 Gy to 0 Gy) [16]. Further, beyond dosimetric values, that study [16] showed that PBT improvement translated to a statistically and clinically significant reduction in toxicity, especially in terms of grade 2 acute toxicities for dysgeusia (6% versus 65%, *P* < .001), mucositis (17% versus 52%, *P* = .02), and nausea (11% versus 57%, *P* = .003) relative to IMRT. Although the reductions in mucosal toxicities and dysgeusia are impressive, also notable is the improvement in nausea, which is likely influenced by less radiation emetogenesis secondary to the marked reduction in the dose to the brainstem and area postrema [17]. In terms of the contralateral salivary glands, which can already be kept to low doses (< 10 Gy) in IMRT, the question as to whether further reduction in dose translates to meaningful clinical benefit is valid; however, as a general rule, the lower the mean dose to the gland, the better the function, as supported by mathematical models [18]. Recently, animal models have suggested that even low doses to high-density stem cell regions of the salivary gland pose consequences to tissue function [19]. Finally, the rates of xerostomia in our study were low, suggesting a likely benefit.

Beyond the sparing of organs at risk and given that many patients with major SGCs are young adults (22% [n = 16] in our study were younger than 40 years), the theoretical reduction of secondary malignancy risk achieved by less integral dose may be clinically significant. Although initially a cause for concern, the risks of neutron contamination seem to be especially low in patients receiving IMPT [20]. Finally, for patients undergoing reirradiation (or those who may require reirradiation in the future), the significant reduction in brainstem and spinal cord dose (about 0.5 GyRBE in the current study) make treatments that would previously exceed annual lifetime tolerances possible and may help to mitigate side effects. In our cohort, 9 patients (12%) were treated who had undergone prior radiation, and they tolerated re-treatment well. With future advancements in treatment optimization, a further benefit with IMPT may come in the form of optimizing plans based on linear energy transfer and RBE, which, by altering beamlet intensity (increasing the weighting of low-energy beams), the linear energy transfer can be elevated within a target and still maintain the same physical dose [9, 21]. That would be ideal for SGCs, given the propensity for certain histologic subtypes (ie, adenoid cystic carcinoma) to harbor resistant cancer stem cells and may further improve the therapeutic index of PBT [22].

The strongest evidence supporting PBT for major SGCs in terms of toxicity lower than historically achieved with IMRT is a recently published review [23] of patients undergoing PBT for major SGCs with curative intent from a multicenter registry of patients enrolled on the Proton Collaborative Group REG001-09 trial (NCT01255748). That study was designed to capture outcomes of PBT but without mandating delivery approach or planning (patients treated per institutional standards of care). In that study, most patients (n = 70) received uniform scanning (PSPT), whereas one third (n = 35) received pencil-beam scanning (IMPT). Tumor control, late toxicities, and dosimetric data were not reported. Although direct comparsons of retrospective studies are challenging, and more patients in our cohort received concurrent chemotherapy (36% versus 20%), rates of grade 2 or higher acute toxicity were comparable in our study for mucositis (14% versus 13%), dysphagia (14% versus 11%), dysgeusia (7% versus 5%), and xerostomia (3% versus 8%). Finally, several other institutional studies of PBT have all reported low rates of mucosal toxicity [24, 25].

Rates of acute dermatitis seem to be high when PBT is used to treat SGCs. Most patients in this report (67, or 93%) experienced grade 2 or higher dermatitis, and 21% (n = 15) experienced grade 3 dermatitis. Secondary to the nature of treatment of the submandibular and parotid region, in which the target remains close to the skin and often includes the postoperative scar, skin sparing is rarely possible without compromising disease control. For example, in the prospective COSTAR trial, rates of grade ≥ 2 acute dermatitis after IMRT were as high as 78% [6]. Given that dermatitis is clinically expected, it should not be surprising that rates are also high for PBT. Indeed, 100% of patients in the Memorial Sloan Kettering Cancer Center study had grade > 2 dermatitis after PSPT [16], and in our study and the Proton Collaborative Group experience [23], dermatitis accounted for most acute grade 2 and 3 toxicities, despite the use of IMPT for many patients. We used stringent criteria for dermatitis grading, which included medical photography to track the evolution and resolution of acute dermatitis per CTCAEv4 grading, rather than relying entirely on treatment assessment reports to minimize underreporting (Figure 3). One inherent benefit with higher physical skin dose is the elimination of the bolus, which helps to reduce the complexity of simulation and treatment setup. Finally, skin toxicity with PBT has been reported for other disease sites and is not unique to the HN. For example, DeCesaris et al [26] found rates of grade 2 radiation dermatitis as high as 70% in patients undergoing proton versus photon treatment for breast cancer. However, in contrast to breast cancer, most patients with HN cancer stand to benefit greatly from the reduction of mucosal toxicity, dysgeusia, nausea, and fatigue, despite the higher rates of acute dermatitis, which, in our cohort did not cause treatment delay or discontinuation. Further, despite the high rate of dermatitis, grade 2 or greater pain was only seen in 26% (n = 19) of the patients and only 1 patient (1%) had grade 3 pain.

Although it is assumed that disease control with PBT would be similar to that with IMRT-based treatment, data are still needed to confirm those assumptions. In our study, the local control rate at 2 years was exceptionally high at 96% (n = 69), and chronic toxicities were rare (<5%) and nonsevere (no grade 3 toxicities) (see Table 3). Although it is not entirely appropriate to compare late toxicities from this study to those from a prospective randomized control trial with double the length of follow-up, it is at least reassuring that our rate of reported chronic toxicity thus far seems to be well below that in the IMRT arm of the COSTAR trial [6], in which rates of late grade 2 or higher toxicities were as high as 44% for tinnitus, 36% for hearing loss, and 22% for xerostomia. Of the 2 patients (3%) in our cohort who developed grade 1 or grade 2 osteoradionecrosis, both were at higher risk because one was undergoing reirradiation and the other was an octogenarian.

### Limitations

Limitations of this study include its retrospective nature (despite prospectively tracked acute toxicity), the mixed tumor histologies, and the single-center design, which limit generalizability. Also, most patients in this study had parotid tumors, making our data less applicable to patients with submandibular gland tumors. Further, despite our best efforts to capture any treatment-related late toxicities, these were not prospectively tracked and may have been underreported in some cases, particularly for patients whose follow-up care was received elsewhere. All efforts were made to follow up with patients not directly seen by phone and mail and to acquire outside records with patient permission. Ultimately, what is needed is a prospective comparison with IMRT to definitely assess for a clinically meaningful improvement with the use of PBT; fortunately, this is being examined in at least one phase II trial (NCT02923570) of PBT versus IMRT for a nonmucosal target, unilateral HN radiation, in which the primary endpoint is grade ≥ 2 acute mucositis. The strengths of the current study include prospective acute toxicity reporting, medical photography for dermatitis evaluation, reporting of dosimetric information, and standardization of treatment planning across all patients.

### Conclusions

Our findings expand on the emerging literature supporting the use of PBT for major SGC, and to our knowledge this report includes the largest sample of patients to have been treated with IMPT. Further, few studies have reported on local control and late toxicity, and our early findings seem promising. Our experience suggests that PBT for the treatment of major SGC manifests in low rates of acute and chronic toxicity and maintains high rates of local and regional control.

## References

[1] Lin HH, Limesand KH, Ann DK (2018). Current state of knowledge on salivary gland cancers. *Crit Rev Oncog*.

[2] Orlandi E, Iacovelli NA, Bonora M, Cavallo A, Fossati P (2016). Salivary gland—photon beam and particle radiotherapy: present and future [published online ahead of print July 6, 2016]. *Oral Oncol*.

[3] Terhaard CHJ, Lubsen H, Rasch CRN, Levendag PC, Kaanders HHÀM, Tjho-Heslinga RE, van Den Ende PLA, Burlage F (2005). The role of radiotherapy in the treatment of malignant salivary gland tumors. *Int J Radiat Oncol*.

[4] Mahmood U, Koshy M, Goloubeva O, Suntharalingam M (2011). Adjuvant radiation therapy for high-grade and/or locally advanced major salivary gland tumors. *Arch Otolaryngol Neck Surg*.

[5] Schoenfeld JD, Sher DJ, Norris CM, Haddad RI, Posner MR, Balboni TA, Tishler RB (2012). Salivary gland tumors treated with adjuvant intensity-modulated radiotherapy with or without concurrent chemotherapy. *Int J Radiat Oncol Biol Phys*.

[6] Nutting CM, Morden JP, Beasley M, Bhide S, Cook A, Winton E De, Emson M, Evans M, Fresco L, Gollins S, Gujral D, Harrington K, Joseph M, Lemon C, Luxon L, van den Blink Q, Mendes R, Miah A, Newbold K, Prestwich R, Robinson M, Sanghera P, Simpson J, Sivaramalingam M, Srihari NN, Sydenham M, Wells E, Witts S, Hall E, for the COSTAR Investigators (2018). Results of a multicentre randomised controlled trial of cochlear-sparing intensity-modulated radiotherapy versus conventional radiotherapy in patients with parotid cancer (COSTAR; CRUK/08/004). *Eur J Cancer*.

[7] Cubillos-Mesías M, Baumann M, Troost EGC, Lohaus F, Löck S, Richter C, Stützer K (2017). Impact of robust treatment planning on single- and multi-field optimized plans for proton beam therapy of unilateral head and neck target volumes. *Radiat Oncol*.

[8] Frank SJ, Cox JD, Gillin M, Mohan R, Garden AS, Rosenthal DI, Gunn GB, Weber RS, Kies MS, Lewin JS, Munsell MF, Palmer MB, Sahoo N, Zhang X, Liu W, Zhu XR (2014). Multifield optimization intensity modulated proton therapy for head and neck tumors: a translation to practice. *Int J Radiat Oncol Biol Phys*.

[9] Ma D, Bronk L, Kerr M, Sobieski M, Chen M, Geng C, Yiu J, Wang X, Sahoo N, Cao W, Zhang X, Stephan C, Mohan R, Grosshans DR, Guan F (2020). Exploring the advantages of intensity-modulated proton therapy: experimental validation of biological effects using two different beam intensity-modulation patterns. *Sci Rep*.

[10] Lassche G, van Boxtel W, Ligtenberg MJL, van Engen-van Grunsven ACH, van Herpen CML (2019). Advances and challenges in precision medicine in salivary gland cancer. *Cancer Treat Rev*.

[11] Romesser PB, Cahlon O, Scher ED, Hug EB, Sine K, DeSelm C, Fox JL, Mah D, Garg MK, Han-Chih Chang J, Lee NY (2016). Proton beam reirradiation for recurrent head and neck cancer: multi-institutional report on feasibility and early outcomes. *Int J Radiat Oncol Biol Phys*.

[12] Seidensaal K, Ben Harrabi S, Uhl M, Debus J (2020). Re-irradiation with protons or heavy ions with focus on head and neck, skull base and brain malignancies. *Br J Radiol*.

[13] Edge S, Byrd D, Compton C, Hess KR, Sullivan DC, Jessup JM, Brierley JD, Gaspar LE, Schilsky RL, Balch CM, Winchester DP, Asare EA, Madera M, Gress DM, Vega LM (2018). American Joint Committee on Cancer. *AJCC Cancer Staging Manual*.

[14] Mohamed ASR, Rosenthal DI, Awan MJ, Garden AS, Kocak-Uzel E, Belal AM, El-Gowily AG, Phan J, Beadle BM, Gunn GB, Fuller CD (2016). Methodology for analysis and reporting patterns of failure in the era of IMRT: head and neck cancer applications. *Radiat Oncol*.

[15] Brouwer CL, Steenbakkers RJHM, Bourhis J, Budach W, Grau C, Grégoire V, van Herk M, Lee A, Maingon P, Nutting C, O'Sullivan B, Porceddu SV, Rosenthal DI, Sijtsema NM, Langendijk JA (2015). CT-based delineation of organs at risk in the head and neck region: DAHANCA, EORTC, GORTEC, HKNPCSG, NCIC CTG, NCRI, NRG Oncology and TROG consensus guidelines. *Radiother Oncol*.

[16] Romesser PB, Cahlon O, Scher E, Zhou Y, Berry SL, Rybkin A, Sine KM, Tang S, Sherman EJ, Wong R, Lee NY (2016). Proton beam radiation therapy results in significantly reduced toxicity compared with intensity-modulated radiation therapy for head and neck tumors that require ipsilateral radiation. *Radiother Oncol*.

[17] Kocak-Uzel E, Gunn GB, Colen RR, Kantor ME, Mohamed ASR, Schoultz-Henley S, Mavroidis P, Frank SJ, Garden AS, Beadle BM, Morrison WH, Phan J, Rosenthal DI, Fuller CD (2014). Beam path toxicity in candidate organs-at-risk: assessment of radiation emetogenesis for patients receiving head and neck intensity modulated radiotherapy. *Radiother Oncol*.

[18] Deasy JO, Moiseenko V, Marks L, Chao KSC, Nam J, Eisbruch A (2010). Radiotherapy dose-volume effects on salivary gland function. *Int J Radiat Oncol Biol Phys*.

[19] Nagle PW, Hosper NA, Barazzuol L, Jellema AL, Baanstra M, Van Goethem MJ, Brandenburg S, Giesen U, Langendijk JA, Van Luijk P, Coppes RP (2018). Lack of DNA damage response at low radiation doses in adult stem cells contributes to organ dysfunction. *Clin Cancer Res*.

[20] Schneider U, Hälg R (2015). The impact of neutrons in clinical proton therapy. *Front Oncol*.

[21] Konings K, Vandevoorde C, Baselet B, Baatout S, Moreels M (2020). Combination therapy with charged particles and molecular targeting: a promising avenue to overcome radioresistance. *Front Oncol*.

[22] Wang L, Han S, Zhu J, Wang X, Li Y, Wang Z, Lin E, Wang X, Molkentine DP, Blanchard P, Yang Y, Zhang R, Sahoo N, Gillin M, Zhu XR, Zhang X, Myers JN, Frank SJ (2019). Proton versus photon radiation-induced cell death in head and neck cancer cells. *Head Neck*.

[23] Chuong M, Bryant J, Hartsell W, Larson G, Badiyan S, Laramore GE, Katz S, Tsai H, Vargas C (2020). Minimal acute toxicity from proton beam therapy for major salivary gland cancer. *Acta Oncol*.

[24] Dagan R, Bryant CM, Bradley JA, Indelicato DJ, Rutenberg M, Rotondo R, Morris CG, Mendenhall WM (2016). A prospective evaluation of acute toxicity from proton therapy for targets of the parotid region. *Int J Part Ther*.

[25] Grant SR, Grosshans DR, Bilton SD, Garcia JA, Amin M, Chambers MS, McGovern SL, McAleer MF, Morrison MH, Huh WW, Kupferman ME, Mahajan A (2015). Proton versus conventional radiotherapy for pediatric salivary gland tumors: acute toxicity and dosimetric characteristics. *Radiother Oncol*.

[26] DeCesaris CM, Rice SR, Bentzen SM, Jatczak J, Mishra MV, Nichols EM (2019). Quantification of acute skin toxicities in patients with breast cancer undergoing adjuvant proton versus photon radiation therapy: a single institutional experience. *Int J Radiat Oncol Biol Phys*.

